# Increased A-to-I RNA editing in atherosclerosis and cardiomyopathies

**DOI:** 10.1371/journal.pcbi.1010923

**Published:** 2023-04-10

**Authors:** Tomer D. Mann, Eli Kopel, Eli Eisenberg, Erez Y. Levanon

**Affiliations:** 1 Mina and Everard Goodman Faculty of Life Sciences, Bar-Ilan University, Ramat Gan, Israel; 2 Tel Aviv Sourasky Medical Center, Tel Aviv, Israel; 3 Raymond and Beverly Sackler School of Physics and Astronomy and Sagol School of Neuroscience, Tel Aviv, University, Tel Aviv, Israel; 4 The Institute of Nanotechnology and Advanced Materials, Bar-Ilan University, Ramat Gan, Israel; Florida International University, UNITED STATES

## Abstract

Adenosine-to-inosine RNA editing is essential to prevent undesired immune activation. This diverse process alters the genetic content of the RNA and may recode proteins, change splice sites and miRNA targets, and mimic genomic mutations. Recent studies have associated or implicated aberrant editing with pathological conditions, including cancer, autoimmune diseases, and neurological and psychiatric conditions. RNA editing patterns in cardiovascular tissues have not been investigated systematically so far, and little is known about its potential role in cardiac diseases. Some hints suggest robust editing in this system, including the fact that ADARB1 (ADAR2), the main coding-sequence editor, is most highly expressed in these tissues. Here we characterized RNA editing in the heart and arteries and examined a contributory role to the development of atherosclerosis and two structural heart diseases -Ischemic and Dilated Cardiomyopathies. Analyzing hundreds of RNA-seq samples taken from the heart and arteries of cardiac patients and controls, we find that global editing, alongside inflammatory gene expression, is increased in patients with atherosclerosis, cardiomyopathies, and heart failure. We describe a single recoding editing site and suggest it as a target for focused research. This recoding editing site in the IGFBP7 gene is one of the only evolutionary conserved sites between mammals, and we found it exhibits consistently increased levels of editing in these patients. Our findings reveal that RNA editing is abundant in arteries and is elevated in several key cardiovascular conditions. They thus provide a roadmap for basic and translational research of RNA as a mediator of atherosclerosis and non-genetic cardiomyopathies.

## Author summary

The human genetic code is highly preserved, yet RNA editing is a process that changes it in RNA molecules. This may alter the resultant proteins or the properties of the RNA strands themselves, leading to changes in their special structure and affecting their interaction with the immune system. Unedited RNA may be highly immunogenic, and studies of recent years demonstrated that dysregulated editing causes an inflammatory response. We show that human RNA editing is especially active in the arteries and investigate its role in cardiovascular diseases such as atherosclerosis and cardiomyopathies. These diseases have an underlying inflammatory component that eventually leads to the loss of normal structure and function. We show that RNA editing is increased in patients and draw a line connecting it with a concomitant increase in inflammatory pathways. We also detected specific coding editing sites, mainly in the IGFBP7 gene, where aberrant editing is present, resulting in an altered protein product. We delineate these changes and suggest they may contribute to or are a marker of the underlying pathology. Our findings indicate that RNA editing is a key player in several cardiovascular diseases.

## Introduction

RNA editing is an endogenous post-transcriptional process, catalyzed by the Adenosine deaminases acting on RNA (ADAR1 and ADAR 2) enzymes. ADAR1 has 2 isoforms, p110 and *p150* which is interferon- inducible. As a result of editing, the RNA sequence is modified from its genomic blueprint, and genomically-encoded adenosines on the RNA molecule are deaminated into inosines [1,2].

Editing occurs in both coding and non-coding sequences of the genome. Editing of a coding sequence may result in amino-acid substitution in the protein product ("recoding"), similar to genomic mutations. Some recoding events have an enormous functional impact. For example, recoding of the Q/R site within the Glutamate ionotropic receptor AMPA type subunit 2 (GRIA2) gene is essential to prevent an inborn fatal phenotype in mice [3,4], and editing of the Antizyme inhibitor 1 (AZIN1) site induces an S/G change, which drives the development of hepatocellular carcinoma [5].

However, virtually all the editing occurs in non-coding repetitive elements, e.g., the human Alu sequences (Alu editing). The current view is that the primordial role of RNA editing is to prevent the identification of endogenous double-stranded RNA structures by the innate immune system. Such structures can be formed when two repetitive *Alu* elements reside close to one another in the same RNA transcript but with the opposite orientation. Double-stranded RNA molecules are potent immune stimulators that the intracellular sensor melanoma differentiation-associated protein 5 (MDA5) and mitochondrial antiviral signaling protein (MAVS) recognize [6].

Editing introduces mismatches between the two RNA strands and disrupts otherwise perfect complementation, the hallmark of viruses [7–9]. This enables escape from immune system activation (S1 Fig in the Supplementary Material). The association between RNA editing and disease has been studied in recent years, particularly in cancer, neurology, and autoimmunity [5,7,10–12]. In cancer, inhibition of ADAR augments the effects of checkpoint inhibitors and is under development as a drug [13].

Exceptionally high levels of A-to-I editing were demonstrated in arteries [14–16], and this observation calls for a better understanding of the cardiovascular (CV) editome in homeostasis and disease. We chose to focus on atherosclerotic cardiovascular disease (ASCVD) and several key cardiomyopathies (CMPs) to explore the possible pathogenic role of disturbed RNA editing.

atherosclerotic-related diseases such as ischemic heart disease (IHD) and cardiomyopathy (ICM), are the most common causes of morbidity and mortality in the western world [17]. They are characterized by narrowing of the heart’s vasculature, structural heart changes and reduced cardiac function, and may culminate in sudden cardiac death or terminal heart failure.

It is increasingly appreciated that atherosclerosis is also a result of vascular inflammation driven by the IFN, IL-1-IL-6 pathway. A number of large-scale clinical trials [18,19], demonstrated the benefit of anti-inflammatory treatments in atherosclerosis, supporting the inflammatory hypothesis. Current evidence suggests that myocardial remodeling occurs in response to ischemia, and inflammation may therefore contribute to ischemic cardiomyopathy formation, thereby propagating atherosclerosis. In addition, inflammation also mediates insult-related repair in ischemic cardiomyopathy, dilated cardiomyopathy, and other cardiomyopathies, since pathological remodeling and scar formation are regulated by inflammatory signals. Accordingly, in parallel to studying the genetic factors underlying atherosclerosis and coronary artery disease, an understanding of the molecular mechanisms that contribute to vascular inflammation may provide additional actionable targets for clinical treatments.

A very recent, pivotal work by Li et al [20] has demonstrated that coronary artery disease (CAD) is heavily influenced by editing and that a genetic tendency to under-edit immunogenic *Alus* is associated with heightened interferon activity and disease prevalence. Common genetic variants that are associated with RNA editing were recognized to be enriched in GWAS signals of autoimmune and immune-related diseases, including CAD, and accounted for a high fraction of disease heritability.

We hypothesized that since RNA editing is associated with inflammation, aberrant editing might contribute to the development of either atherosclerosis or non-genetic cardiomyopathies. We additionally speculated that a single editing disruption at a critical recoding site may adversely affect cellular function and lead to a disease, as reported with FLNA editing and cardiomyopathy [14].

Here we analyzed RNA sequencing data from the GTEx project, encompassing thousands of human samples, as well as additional datasets of cardiomyopathy patients. We observed an increase in both intronic and recoding editing in atherosclerosis patients, with a particular increase in Insulin-like growth factor binding protein 7 (IGFBP7) gene editing. Similarly, we observed increases in intronic editing in patients with various types of cardiomyopathies. We have confirmed that these pathologies are accompanied by increased expression of inflammatory genes [21]. Finally, we discuss the possibility that interferon-related inflammation leads to enhanced ADAR1 *p150* expression, resulting in elevated editing in cardiovascular pathologies.

## Methods

### Construction of the GTEx patient groups

GTEx patients were classified according to their medical records and the medical examiners’ diagnoses. The atherosclerosis group contained all patients who died from or had a history of myocardial infarction or ischemic heart disease. The cerebrovascular group contained patients who either died from or had a history of cerebrovascular accident (CVA), intracranial hemorrhage (ICH), or cerebral aneurysm. The control group contained all other individuals not included in the atherosclerosis and cerebrovascular groups, with the exception of patients who died from an overwhelming infection who were removed from the analysis.

### RNA-seq data preprocessing and quality control

Transcriptomic data quality was evaluated using the FastQC quality control tool version 0.10.1 [22]. The reads were mapped uniquely (outFilterMultimapNmax = 1) to the human reference genome version GRCh38/ hg38 using STAR aligner version 2.5.2b [23].

### Gene expression quantification

We used Salmon tool version 0.11.2 to quantify the transcript levels of the RNA-seq data in units of transcripts per million (TPM) [24] using default parameters and indexed human genome version GRCh38/hg38. We then converted the output from transcript to gene-level using “tximport” R package version 1.12.3.

### Global RNA editing levels in *Alu* repetitive elements

Most editing in humans takes place within *Alu* repeats [25]. To measure the editing in *Alu* elements, we used the Alu Editing Index (AEI) method version 1.0 [15]. Briefly, the global RNA-editing index was calculated as the ratio of guanosine at all the adenosine positions in *Alu* over adenosine and guanosine at the same genomic locus. A higher index indicates a greater percentage of editing activity in a sample. To clean the editing signal further, we analyzed an additional subset of 3,031 genomic *Alus* that are located within 3’ UTR of genes, have a minimum length of 250bp, and have an oppositely oriented *Alu* on the same 3’ UTR.

### RNA editing quantification in coding editing sites

We used the REDIToolKnown script to measure A-to-I RNA editing levels in coding editing sites [26]. The script quantifies the editing levels in a set of 314 previously defined exonic editing sites [27]. The parameters we used allowed a minimum of one read supporting the variation (−v 1), minimum 0.001 editing frequency (−n 0.001), exploring one base near splice junction (−r 1), minimum one read coverage, and trimming of five bases at both ends of the reads (−T 5–5). For each patient we first calculated the Coding Editing Index (CEI), which provides a global measure of editing in coding sequences. For this purpose, we calculated the ratio of the total number of A-to-G mismatches at all the 314 sites together over the total number of reads aligned to all of these positions. Then we calculated the editing level of each of the 314 sites separately. Only sites with at least ten supporting reads were included in the analyses.

### Interferon Signature (ISG)

To evaluate IFN signaling activity modifications, we first calculated the gene expression levels of the interferon genes using the Salmon tool version 0.11.2 [24] with default parameters. The output was then used as input for the ISG pipeline, which uses a 38-gene signature to provide an ISG score [21].

### Ribosome profiling preprocess

We used “Trimmomatic” (version 0.33) with the parameters: -phred33 LEADING:3 TRAILING:3 MINLEN:20 to remove the Illumina trueseq adapters from each ribo-seq fastq format file and a “bowtie” aligner (version 1.2.3) with default parameters to index the human hg38 genome and align fastq files to SAM format. To avoid contamination, we used “FastqScreen” (version 0.14.0) to filter out reads that aligned to the rRNA transcriptome. In order to optimize alignment, we added the -v 2 parameter, which permits a maximum of 2 mismatches in each read while aligning. We sorted the SAM with “Picard” (version 2.5.0) SortSam with the parameter SORT_ORDER = coordinate. We indexed the Bam file using Picard BuildBamIndex and default parameters.

### Statistics and figures

All values in graphs are expressed as the mean ± SEM. Normality was tested with the Shapiro-Wilk normality test. If the data exhibited a normal distribution, we performed pairwise testing with two-sided, unpaired, Student’s t-test, and multiple group comparisons with a 2-way ANOVA followed by Tukey post-test. For non-normally distributed data or N < 30, we performed pairwise testing using the two-sided non-parametric Mann-Whitney U test with multiple group comparisons by the non-parametric Kruskal-Wallis test, followed by Dunn’s post-test. Corrections for multiple comparisons were made by the Benjamini–Hochberg false discovery rate (FDR) multiple testing procedure.

All tests and analyses were performed using “R” version 3.5.3 (R Core Team 2014) and Rstudio, an integrated development environment for R, version 1.3.1093. Figures were drawn with R, Microsoft Excel, and Adobe Illustrator (version 24.0.1).

## Results

### RNA editing is exceptionally high in arteries

A major resource of human RNA-seq data is the GTEx cohort [28], which includes thousands of samples from donors with various backgrounds and medical conditions.

A previous analysis of the extensive GTEx dataset [29], which included RNA sequencing of 8848 samples and 47 tissues, showed *Alu* editing was the highest in the arteries (GTEx tissue types: Aorta, Coronary arteries, Tibial artery), but not in the heart (Left Ventricle: LV, and Left Atrial Appendage: LAA(15). However, the scope of editing in coding sequences is unknown.

**Fig 1:**
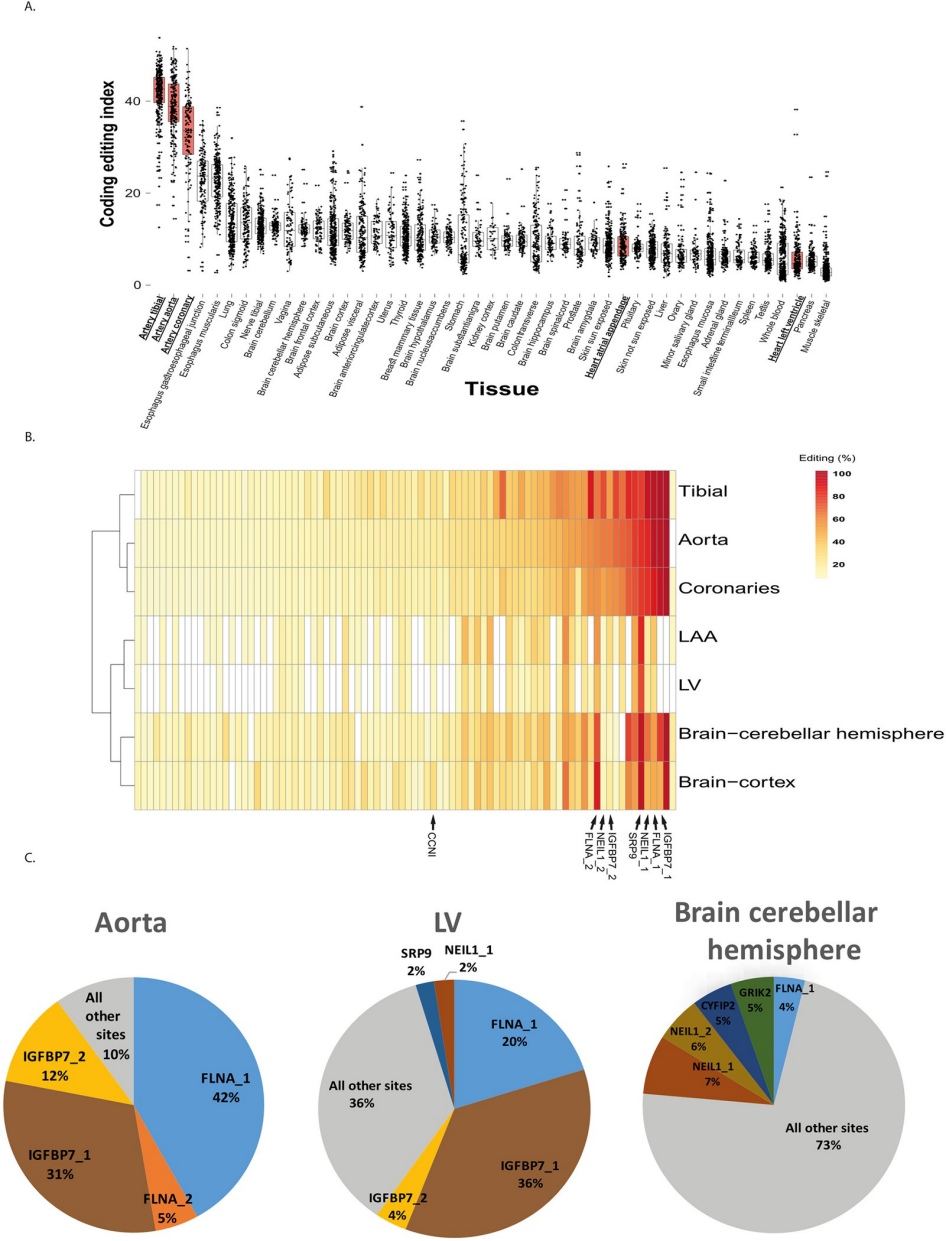
RNA editing in coding sequences is exceptionally high in arteries. **(a)** Coding Editing Index of all GTEx samples, presenting the editing level per donor as a weighted average over 314 coding editing sites. **(b)** Clustering tissues by the profile of editing levels for the 314 sites (calculated for pooled GTEx data). Columns correspond to editing sites meeting cutoff values of ≥ 5% editing and expression in at least five tissues. Colors represent the editing percent at each site. The values correspond to the pulled average per site for all samples meeting the specified cutoffs for each site. Arrows indicate the positions of IGFBP7, FLNA, NEIL1, CCN1 and SRP9, the sites where most cardiovascular editing takes place**. (c)** The relative contribution of specific coding sites to the overall recoding activity. Notably, all highly edited sites are recoded (editing causes an amino-acid change) and evolutionarily conserved across mammals—samples from left ventricles of unselected GTEx donors.

Here we analyze the same dataset to compare the level of editing within the coding region across tissues. We extended the previously published Recoding Editing Index [30] to a Coding Editing Index (see Methods), which also includes synonymous editing sites, and found editing in coding areas in the arteries to be the highest of all other 47 tissues, including the brain, which is believed to be the most edited tissue (Fig 1A).

Both the arteries and the brain have highly edited recoding sites. However, unlike the brain, the sites in the arteries are strongly expressed, leading to a much higher number of edited transcripts.

To illustrate this point, we compared the total number of edited transcripts, averaged over donors. Summing over the 314 evaluated coding sites (see Methods), the number of A-to-G mismatches (each representing an editing event) is an order of magnitude larger in artery tissues than in the cerebellar hemisphere, the brain’s most highly edited tissue (11685, 12014, and 9442 transcripts per tissue in the aorta, tibial, and coronary arteries, respectively, vs. 1,700 in the cerebellar hemisphere). In the arteries, the number of recoding events in FLNA transcripts alone (4897, 5582, and 4187 per sample in the aorta, tibial, and coronary arteries, respectively) is considerably larger than the total number of edited transcripts in the brain [14].

Interestingly, the editing profile across all 314 coding sites reveals tissue-specific patterns that largely reflect the anatomy and function of cardiovascular tissues. We ran a PCA based hierarchical clustering analysis and found that arteries cluster separately from the LV and LAA as two distinct groups. Within the arteries, the aorta and coronaries share a similar pattern that is distinct from that of the tibial artery (Fig 1B). This suggests that editing patterns indeed share unique characteristics of each cardiovascular tissue.

An additional difference we discovered between the brain and the cardiovascular tissues is that cardiovascular editing is highly centralized- i.e., a small number of sites account for the majority of recoding editing. To illustrate this point, we inspected the distribution of editing in specific genes. We found that the five most edited targets in cardiovascular tissues are IGFBP7, FLNA, NEIL1, CCNI, and SRP9. Together, these five sites account for 92%, 91%, 90%, 64%, and 68% of all coding editing activity in the aorta, coronary arteries, tibial artery, LV, and LAA, respectively. The markedly different behavior of recoding activity between the tissues is due to both elevated expression and higher editing levels of the abovementioned few recoding sites. In contrast, the top five edited sites in the cerebellar hemisphere account for only 29% of the editing activity (Fig 1C).

Recoding in cardiovascular tissues is focused on a few editing sites, in stark contrast to the complex recoding profile in the brain. This difference may be partly attributed to differences in the cellular composition of each tissue. Brain tissues exhibit diverse cellular populations [31], while the majority of heart cells are cardiomyocytes and cardiac fibroblasts [32]. This leads to a homogenous editing profile across cardiovascular tissues, as opposed to the heterogeneous structure of the brain.

Notably, mutations and altered expression of IGFBP7 and FLNA have been observed in various cardiovascular diseases [14,33–39].

### RNA editing in *Alu* sequences increases in atherosclerosis and cardiomyopathies

A medical record of each GTEx donor is available upon request. We used this information to further analyze the GTEx cohort and divided its donors into three groups of interest: patients with atherosclerosis (n = 104), patients with cerebrovascular disease (CVAs, aneurysms, and ICH; n = 145), and controls (n = 228, S1 Table in the Supplementary Material; Methods).

**Fig 2:**
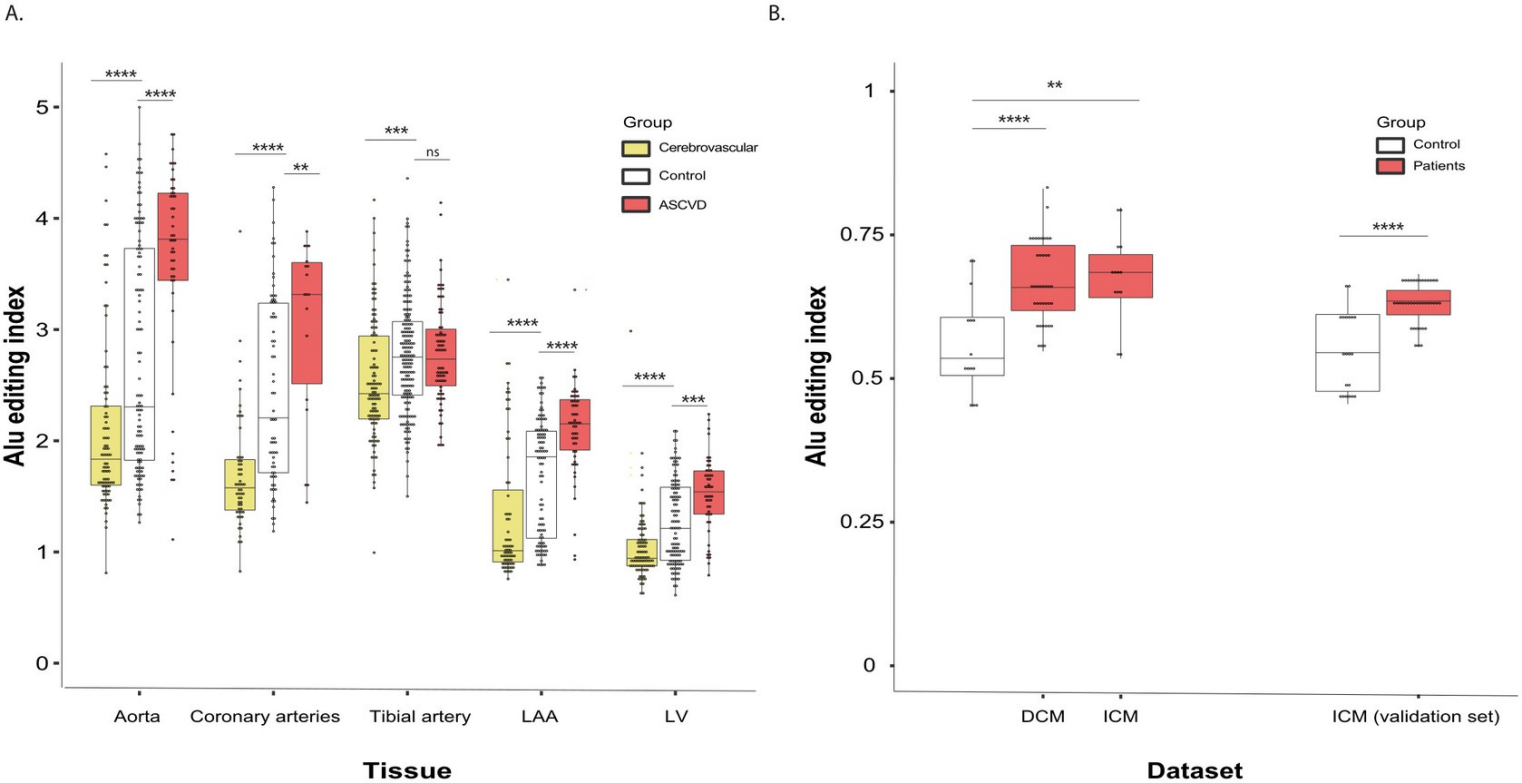
RNA-editing in Alu sequences in atherosclerosis and cardiomyopathies. AEI demonstrates consistently increased editing levels of all *Alu* sequences in **(a)** atherosclerosis (ASCVD: red; controls: White; Cerebrovascular: yellow) and **(b)** CMPs (Patients: red; Controls: white) patients, and hypo-editing in cerebrovascular patients. The DCM and ICM groups are compared to the same control group. Note that due to differences in read length, the nominal index values cannot be compared across the two panels. The exact p-values are detailed in Tables 1 and 2.

In addition to the GTEx cohort, we analyzed other datasets including ventricular samples from three different cardiac pathologies [40] (S2 Table in the Supplementary Material) with matching healthy controls. We found that atherosclerosis patients demonstrate a significant and consistent increase in the *Alu* editing in all cardiovascular tissues except the tibial artery (Fig 2A). Consistent with this observation, ischemic cardiomyopathy patients also demonstrated increased *Alu* editing (Fig 2B), as did those with non-ischemic types of cardiomyopathies (i.e. dilated cardiomyopathy) (Table 1 and Fig 2B). In contrast, patients with cerebrovascular disease demonstrated a decrease in *Alu* editing across all cardiovascular tissues (Table 2 and Fig 2A).

**Table 1:** AEI and CEI in ischemic and dilated cardiomyopathies (ICM, DCM,mean ± SEM). Red upward arrows represent significantly increased editing levels compared with controls (Wilcoxon rank-sum test). Note that ICM and DCM are both compared to the same control group.

		Artery aorta	Artery tibial	Artery coronary	Heart LV	Heart LAA
AEI	ASCVD	3.59 ± 0.14 (n = 47, p = 1.3e-5) **↑**	2.78 ± 0.05 (n = 76, p = 0.94)	3.04 ± 0.19 (n = 18, p = 0.008) **↑**	1.51 ± 0.05 (n = 48, p = 3e-4) **↑**	2.11 ± 0.06 (n = 53, p = 1.1e-5) **↑**
	Cerebrovascular disease	2.1 ± 0.09 (n = 86, p = 6.4e-6) **↓**	2.53 ± 0.05 (n = 108, p = 1e-4) **↓**	1.7 ± 0.07 (n = 56, p = 1.49e-7) **↓**	1.04 ± 0.03 (n = 86, p = 2.7e-5) **↓**	1.33 ± 0.08 (n = 67, p = 5.55e-6) **↓**
	Controls	2.7 ± 0.1 (n = 113)	2.77 ± 0.04 (n = 173)	2.45 ± 0.1 (n = 67)	1.26 ± 0.04 (n = 112)	1.68 ± 0.05 (n = 97)
CEI	ASCVD	42.9 ± 1.0 (n = 46, p = 1.75e-5) **↑**	42.83 ± 0.62 (n = 74, p = 0.19)	35.68 ± 2.21 (n = 17, p = 0.75)	7.92 ± 0.46 (n = 48, p = 4.7e-6) **↑**	10.34 ± 0.49 (n = 53, p = 0.003) **↑**
	Cerebrovascular disease	36.6 ± 0.7 (n = 85, p = 0.02) **↓**	40.9 ± 0.46 (n = 108, p = 0.03) **↓**	29.43 ± 1.25 (n = 56, p = 0.001) **↓**	4.64 ± 0.43 (n = 86, p = 3e-4) **↓**	7.43 ± 0.39 (n = 67, p = 0.002) **↓**
	Controls	38.7 ± 0.6 (n = 113)	41.8 ± 0.4 (n = 173)	34.83 ± 0.8 (n = 67)	5.7 ± 0.34 (n = 112)	8.71 ± 0.33 (n = 94)

**Table 2:** AEI and CEI in cardiac and cerebrovascular patients (mean ± SEM). Note that due to differences in read length, the nominal index values cannot be compared with those in Table 1. The color and direction of the arrows represents the change (upward red, elevated editing levels; downward blue, reduced editing levels) and the significance (Wilcoxon rank-sum test).

		ICM	DCM	ICM (validation)
AEI	Patients	0.68 ± 0.02 (n = 13, p = 0.002) **↑**	0.67 ± 0.01 (n = 37, p = 0.0001) **↑**	0.63 ± 0.01 (n = 20, p = 1.55e-05) **↑**
	Controls	0.56 ± 0.02 (n = 14)	0.56 ± 0.02 (n = 14)	0.55 ± 0.02 (n = 10)
CEI	Patients	4.14 ± 0.59 (n = 13, p = 0.002) **↑**	3.51 ± 0.19 (n = 37, p = 9.72e-06) **↑**	5.71 ± 0.41 (n = 20, p = 1.29e-05) **↑**
	Controls	2.36 ± 0.14 (n = 14)	2.36 ± 0.14 (n = 14)	3.22 ± 0.23 (n = 10)

We have employed a multivariate regression model, including gender and age as additional independent variables, to control for gender and age differences between the groups.

A pathological role for *Alu* editing in atherosclerosis patients related to editing in the 3’ UTR of the CTSS gene has previously been discovered [41]. Inspired by this result, we examined the editing in all *Alu*s within 3’ UTRs and found that, the editing in these *Alu*s increases in atherosclerosis, dilated and ischemic cardiomyopathies, while they decrease in cerebrovascular patients (S2 Fig in the Supplementary Material).

### Interferon-stimulated genes are increased in cardiomyopathies and correlate with elevated ADAR1 expression

The ADAR1 *p150* isoform is interferon-inducible and has been linked to inflammatory processes [42]. We hypothesized that the elevated editing we observed in cardiomyopathies and atherosclerosis patients results from underlying tissue inflammation that mediates the pathological process.

**Fig 3:**
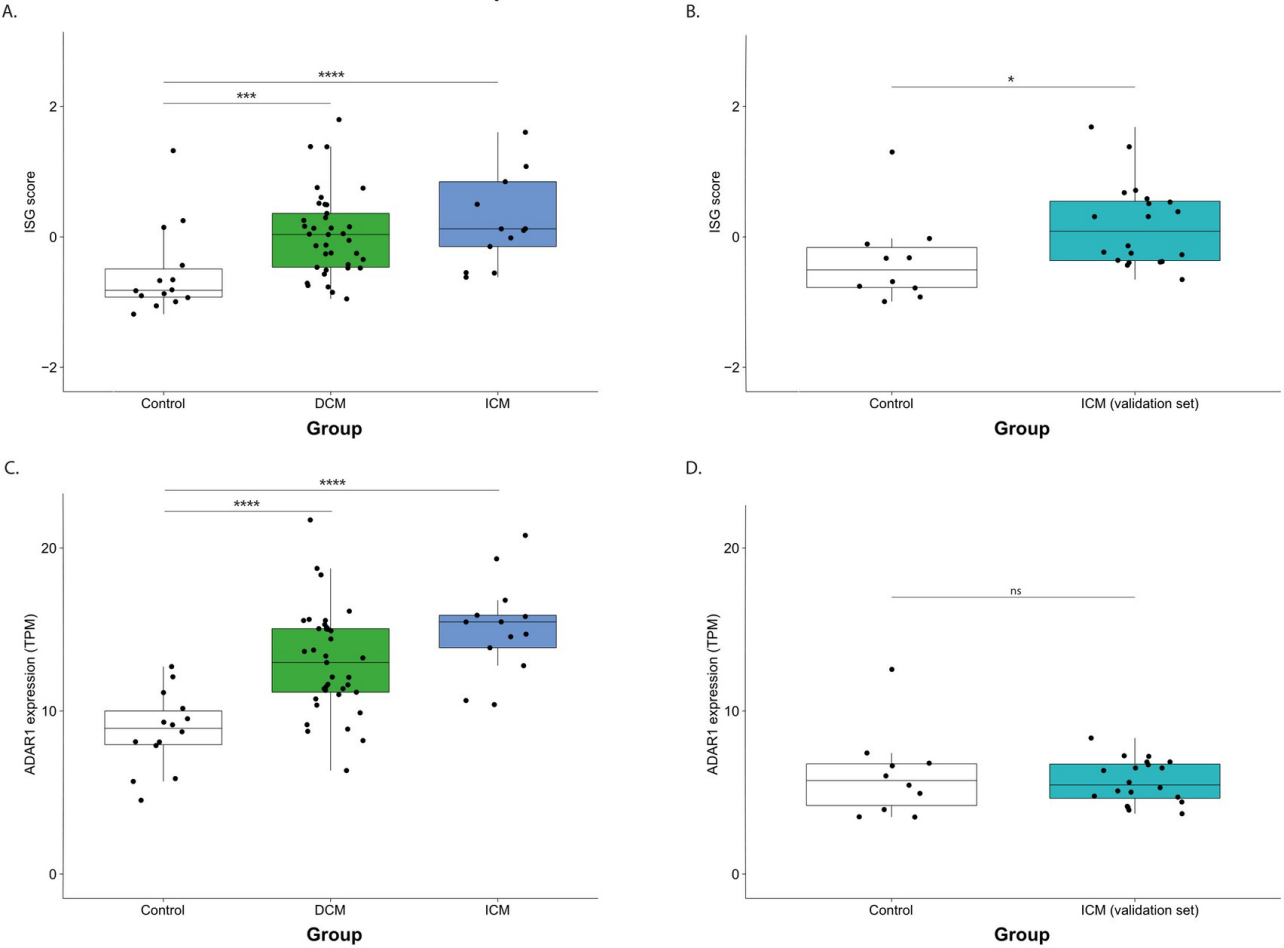
Interferon stimulated genes and ADAR1 expression in CMPs. Interferon Signature (ISG) Score in **(a)** CMPs and **(b)** ICM patients. ADAR1 expression levels in transcript per million units in patients with (c) CMPs and **(d)** ICM. The exact p-values are detailed in Tables 1 and 2.

In order to examine this hypothesis, we measured the ISG score [21], which considers the expression level of 38 genes in the IFN cascade (including ADAR1). The ISG scores were higher in cardiomyopathy patients than in controls (Wilcoxon rank-sum test, p-value = 0.00005, 0.001 for ischemic and dilated cardiomyopathies, respectively, and P = 0.017 for the validation ICM set, Fig 3A and 3B), supporting the association of these pathologies with inflammation. The other GTEx cohorts did not exhibit any significant change in the ISG score, presumably because they are associated with milder heart conditions. Furthermore, the results in Fig 3C and 3D, demonstrate that ADAR1 expression is upregulated in ischemic and dilated cardiomyopathies compared to controls (Wilcoxon rank-sum test, p-value = 0.00002, 0.000003 in dilated and ischemic cardiomyopathies, respectively), with no change in the other cohorts. ADAR2 is not known to be affected by interferon. As expected, we did not detect any change in its expression between patients and controls.

Moreover, we observed a positive linear relationship between ADAR1 expression, and the *Alu* editing and ISG scores in cardiomyopathies samples, which implicates RNA editing in these conditions, and these three parameters exhibit positive pairwise correlations (Spearman, rho = 0.48, 0.58 and 0.45; p-value = 1.5e-06, 1.2e-09 and 5.9e-06, for ADAR1 expression vs. ISG score, ADAR1 vs. *Alu* editing and *Alu* editing vs. ISG, respectively). Linear regression analysis revealed that the *Alu* editing is significantly correlated to both ADAR1 expression and the ISG score, with a positive interaction term (p for interaction = 0.004) (S2 Fig).

### Editing in recoding sites increases in various cardiovascular diseases

**Fig 4:**
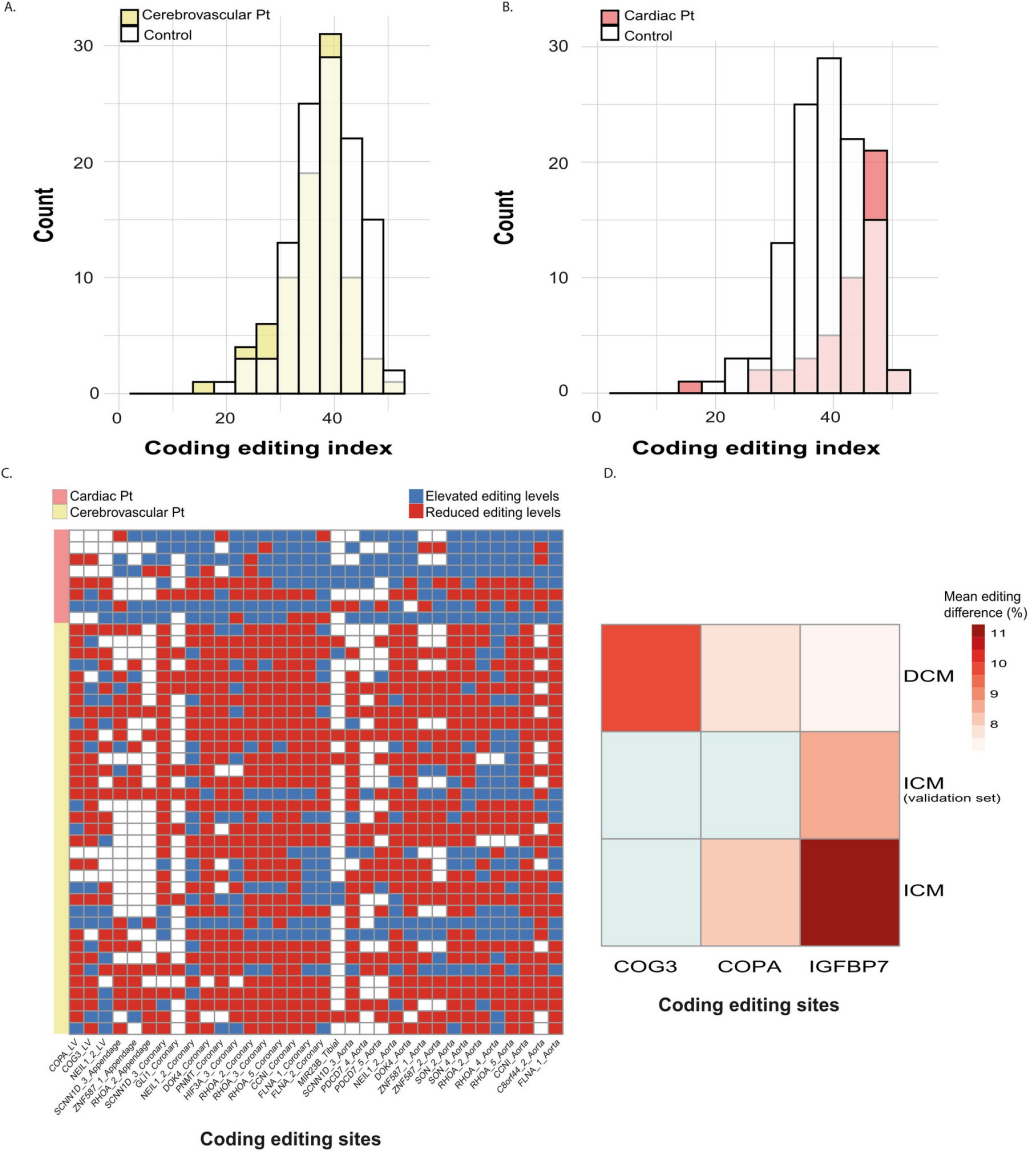
The landscape of coding editing in cardiovascular patients. Coding Editing Index distribution in **(a)** Aortae of cerebrovascular and **(b)** ASCVD patients. **(c)** Heatmap summarizing all significant (FDR cutoff of ≤ 0.05) and meaningful (editing index difference ≥ 5%) coding editing sites in cardiovascular tissues from GTEx data. Columns are editing sites. Rows represent individual samples of cardiovascular tissue taken from donors with either cardiac or cerebral disease. The heatmap presents 139 patients who have information for at least 70 editing sites. We calculated the change in editing levels for each patient in each site by subtracting the control group average at that site. The color represents the direction of change (blue, elevated editing levels; red, reduced editing levels). **(d)** Summary of the significant changes in editing in the cardiomyopathies cohorts. Columns are editing sites that differentiate patients from controls in at least one disease type. Colors represent the mean difference in editing levels compared to controls. Gray cells represent missing data or differences not meeting the significance cutoff. Significance is defined by the Wilcoxon rank-sum test p-values followed by the Benjamini–Hochberg procedure with FDR < 0.05.

While less than 1% of all RNA editing activity occurs in protein-coding sites, such events may have a far-reaching pathological impact. Significant differences in editing at specific coding sites may be of particular interest if they mimic a genetic variant or mutation. We, therefore, focused our coding site analysis on sites that exhibit a meaningful biological change in the editing level (defined as a mean difference of at least 5%) in disease. Analyses were performed separately for each of the cohorts. We found that coding editing was increased in atherosclerotic cardiac diseases and decreased in cerebrovascular diseases, reminiscent of what we observed in *Alu* editing (Fig 4A and 4B). 34 coding sites demonstrated meaningful and significant alterations, with most sites showing increased editing levels in atherosclerosis and conversely- a decrease in editing in cerebrovascular patients, compared with controls (Fig 4C and S3 Table in the Supplementary Material). A recent comprehensive analysis of the GTEx cohort [15], precluded a major influence of baseline characteristics such as sex and age on the editing levels. Hence, the main driver of such differences is likely the existence of a disease.

Although the cohort’s sizes of ICM and DCM were relatively small, we could still detect three differentially edited sites within the IGFBP7, Copper-exporting P-type ATPase (COPA), and Conserved oligomeric Golgi complex subunit 3 (COG3) transcripts that exhibited a substantial increase (Table 3 and Fig 4D). These sites are mainly edited by ADAR2 [43–46]. We did not detect differences in the expression of this enzyme in either disease compared with controls, and thus the source of the differential editing in these sites is unclear. It is worth mentioning that, ideally, the existence of such differences should be discerned in the arteries in ICM, where ADAR2 expression is also notably high. However, only LV samples were available for this cohort.

**Table 3:** RNA-editing levels in specific coding sites in CMPs. Statistical analysis was performed using Wilcoxon rank-sum test followed by the Benjamini–Hochberg procedure for false discovery rate (FDR) for multiple testing (< 0.05).

Site	Gene name	Dataset	Mean control (%)	Mean patient (%)	P. value	FDR
chr4 57110068	IGFBP7	ICM (validation)	18.95 ± 1.63	27.41 ± 1.35	0.00	0.00
chr1 160332454	COPA	ICM	12.95 ± 1.79	21.09 ± 2.78	0.03	0.04
chr4 57110068	IGFBP7	ICM	15.58 ± 1.3	26.86 ± 2.91	0.01	0.02
chr1 160332454	COPA	DCM	12.95 ± 1.79	20.84 ± 1.57	0.01	0.02
chr13 45516236	COG3	DCM	12.23 ± 2.05	21.90 ± 1.98	0.01	0.02
chr4 57110068	IGFBP7	DCM	15.58 ± 1.3	22.71 ± 1.24	0.00	0.01

In order to examine whether the recoded RNA transcripts were in fact being translated into proteins, bearing a potential functional impact, we analyzed ribosome profiling data [47] of left ventricular samples from DCM donors (n = 30), which are enriched for translated transcripts. Measurements of the editing levels at conserved mammalian sites verified the presence of increased editing (S3 Fig in the Supplementary Material) [48–51].

### IGFBP7 editing as a potential diagnostic marker for underlying ischemic disease

Cardiac markers of injury greatly facilitate the management of ischemic and heart failure patients, and novel markers are constantly sought after. IGFBP7 can be sampled from the peripheral blood and serum levels of this protein increase in atherosclerosis and heart failure with preserved ejection fraction and correlate with cardiac function [37,52–55].

Our analysis demonstrates that the editing of IGFBP7 is markedly increased with cardiac or vascular injury (increases in LV editing from 16% to 26% and 26% to 34% in ischemic cardiomyopathy and atherosclerosis, respectively). Integrating both IGFBP7 serum levels and edited isoform fraction may thus further increase its diagnostic performance without the need for sequencing, rendering it an accessible and potentially useful novel clinical marker. This can be done, for instance, by sampling peripheral blood and utilizing specific antibodies for the edited and non-edited versions of IGFBP7.

Considering that our findings suggest the functional relevance of RNA editing to a variety of cardiovascular conditions, we examined how well the tissue editing levels correlate with those of the peripheral blood. We used the GTEx cohort for this purpose since it contains data from numerous tissues. Indeed, the Spearman test demonstrated a strong correlation between the blood Alu editing level and four of the five cardiovascular tissues (rho LV = 0.72; n = 175, LAA = 0.8; n = 183, aorta = 0.79; n = 184, coronaries = 0.62; n = 113, and tibial artery = 0.38; n = 268, and p values = < 2.2e-16, < 2.2e-16, < 2.2e-16, < 2.2e-16, and 3.2e-10, respectively). We next wanted to assess how increased editing translated into a potentially clinically useful test. We tested this by comparing the predictive value of Alu editing and IGFBP7 editing with expression-based levels of common clinical markers of cardiac injury. We found that, in the cardiomyopathies, editing of both *Alu* and IGFBP7 has a predictive value that is comparable to the expression of top heart failure markers, such as troponin subtypes, atrial natriuretic peptide (ANP), and B-type natriuretic peptide (BNP, AUC: ANP = 0.73, BNP = 0.9, Troponin I = 0.92, Troponin T = 0.69, IGFBP7 editing = 0.8, Alu editing = 0.84, S4 Fig in the Supplementary Material). This suggests that the magnitude of editing changes is on the same scale as state-of-the-art cardiac markers.

## Discussion

Advancements in diagnostics and therapeutics of cardiovascular illnesses rely on a deeper understanding of the underlying cellular processes. RNA editing is highly abundant in vascular tissues, is tissue-specific, and participates in pathways that contribute to cardiovascular diseases, such as inflammation and circular RNA regulation. Li et al. recently pointed a spotlight on RNA editing demonstrating it is a major mediator of the genotypic effects on inflammatory diseases [20]., including atherosclerosis. Further investigating the role and mechanisms this process plays in the pathogenesis of common inflammatory-driven diseases and possible ways to intercept them is therefore timely and warranted.

RNA editing appears to interfere with several relevant mechanisms. Jain et al. [14] recognized that aberrant editing at the recoding FLNA site contributes to dilated cardiomyopathy and identified a 50% reduction in editing at this site in patients. Mice lacking the ability to edit the FLNA site develop heart failure and diastolic hypertension. In addition, a strong downregulation of ADAR2 and an increase in ADAR1 expression were observed in patients with congenital heart defects [56].

Here we present for the first time a comprehensive analysis of RNA-editing in the cardiovascular system. We found that the highest activity of editing in both coding and non-coding sequences occurs in the cardiovascular system. Patients with coronary or structural heart disease demonstrated yet higher levels of editing compared with controls. Conversely, editing decreases in patients with cerebral vascular accidents or hemorrhages but no heart disease. These opposing trends may represent differences in etiology (i.e., atherosclerosis and remodeling in cardiac patients versus cardio-embolic strokes and bleeding from aneurysms in cerebrovascular patients), or else the aberrant editing may serve as an etiology.

In recent years, it has become accepted that inflammation plays a central role in conditions such as atherosclerosis and the resulting ischemic cardiomyopathy [57–60]. Although cardiovascular inflammation has been extensively investigated using various methodologies [61,62], key drivers of the inflammatory signals remain elusive. RNA editing is known to take part in the inflammation cascade since ADAR1 *p150* expression is strongly influenced by IFN. Accordingly, we observed a close correlation between inflammation and increased ADAR1 expression and *Alu* editing in ischemic and dilated cardiomyopathies. Interestingly, *Alu* editing is most increased in ICM, where inflammation may mediate both the initial pathology and subsequent scar formation. It is possible that RNA editing represents a new inflammation-driving mechanism that is highly active in the arteries. Importantly, attempts are currently made to both activate and inhibit the editing machinery for therapeutic purposes. This may render RNA editing an actionable target.

It is important to note that RNA editing is present and maybe upregulated even in the absence of inflammation. Alu editing was increased in the GTEx data analysis of patients with atherosclerosis and cerebrovascular disease, despite normal ISG scores. It is possible that the lack of correlation between the ISG score and Alu editing in this cohort is due to the nature of the GTEx population, which comprises mainly deceased donors, and where tissue ischemia and other perimortem changes may affect gene expression and blur an analysis that relies on differential expression. Given this potential caveat, it is noteworthy that the Alu editing in this population still differentiates patients from controls, testifying to the robustness of the measurement. Another possible mechanism by which altered editing promotes structural heart diseases and atherosclerosis is through effects on the formation of circRNA. An increase in RNA editing in intronic regions has been shown to reduce circRNA levels [60]. This reduction may in turn contribute to the development of heart failure, dilated cardiomyopathies, and probably also to atherosclerosis [57–59,63,64]. Aberrant editing may therefore be an initiating factor.

Our results identified several specific coding sites with prominent differences between patients and controls. These recoding changes are enriched for relevant biological processes. For example, in left ventricle samples, we observed an enrichment of genes controlling actin and cytoskeleton polarity, a process that plays a crucial role in cardiomyopathy and heart failure. Similarly, we observed enrichment in atrial cell membrane repolarization regulation in the left atrial appendage -a process that may be involved in arrhythmias and thrombus formation.

Finally, a primary site of interest for cardiovascular editing is within the IGFBP7 gene. This gene is highly expressed in cardiovascular tissues and contains two evolutionarily conserved editing sites. We observed altered editing in the major editing site in atherosclerosis patients. Editing in this site in cardiovascular tissues is exceptionally high, surpassing 95%, and constitutes a sizable proportion of all recoding editing in the heart and arteries. The mechanism by which the observed increase in editing of IGFBP7 influences pathogenesis is unclear. It is possible that editing of this highly expressed cardiovascular gene is a result of the globally increased ADAR1-mediated editing. However, some indirect evidence suggests that this site is edited by ADAR2 and is not so much susceptible to ADAR1 overexpression.

IGFBP7 has already been suggested as a biomarker of atherosclerosis and heart failure [54,55], and mutations in this gene are known to lead to vascular and valvular pathologies [60]. The notion that IGFBP7 could serve as a clinical marker is supported by our finding that in addition to the elevated serum levels seen in patients, the editing levels of this protein are also increased. [65] Editing changes at this site in cardiomyopathy patients are comparable to the elevations in gene expression of top cardiac biomarkers. Taken together, our findings suggest that RNA editing is involved in atherosclerosis and subsequent ischemic, as well as in dilated cardiomyopathies. Further research is warranted to assess the role of this emerging process in the development of these diseases.

### Limitations

The main limitation of our study is its in-silico nature, which naturally precludes the ability to infer causality and mandates further biological experiments.

Another limitation is the use of publicly available data, where complete details on the donors are sometimes lacking, along with a lack of control over the data production process. For example, no genotypic information was available in the cardiomyopathies datasets. This limitation is common in bioinformatical analysis in general, and we addressed this issue by carefully selecting datasets with high-quality and maximal annotations.

We made every effort to use reproducible, meticulous analysis of the data and to use well-established and consistent statistical approaches to maximize the robustness of our findings.

## Conclusion

RNA editing in both coding and non-coding regions is increased in atherosclerosis, ischemic, and dilated cardiomyopathies. It is partially correlated with increased inflammatory signals and thus might be involved in maladaptive inflammatory remodeling. RNA editing is not fully explained by increased inflammation and may represent a discrete additional process.

## Supporting information

S1 Fig RNA editing protects against self-immune attacks. Endogenous dsRNA structures are formed naturally and may activate the inflammation pathway. To prevent false activation of the immune system, ADAR1 disrupts endogenous dsRNA structures by editing adenosines to inosines. Mitochondrial Antiviral Signaling (MAVS); Melanoma Differentiation-Associated protein 5 (MDA5); Interferon Stimulated Genes (ISG); Adenosine Deaminase Acting on RNA 1 (ADAR1).
(JPG)

S2 Fig RNA-editing in Alu sequences within 3’ UTR in ASCVD, CMPs. The AEI demonstrates consistent increased editing levels of all *Alu* sequences in **(a)** ASCVD patients, and hypo-editing in cerebrovascular patients (Two-sided Wilcoxon rank-sum test, p. value = 0.0004, 0.024, 0.005, 0.0002, 0.78 for ASCVD and 1.7e-6, 4.1e-7, 4.1e-5, 8.6e-5, 0.0001 for cerebrovascular in the aorta, coronary arteries, LAA, LV and tibial artery, respectively). Increased editing levels are also observed in **(b)** CMP (Two-sided Wilcoxon rank-sum test, p. value = 4e-06 and 1.1e-05 for DCM and ICM, respectively) and **(c)** ICM (Additional validation set) (Two-sided Wilcoxon rank-sum test, p. value = 0.001). Note that due to differences in read length, the nominal index values cannot be compared between the two panels.
(JPG)

S3 Fig Three-way correlation between the Interferon stimulated gene score (ISG), ADAR1 expression, and the Alu editing index (AEI) in CMPs. Spearman correlation rho = 0.48, 0.58 and 0.45; p-value = 1.5e-06, 1.2e-09 and 5.9e-06, for ADAR1 expression vs. ISG score, ADAR1 vs. Alu editing and Alu editing vs. ISG, respectively). Linear regression analysis revealed that the Alu editing is significantly correlated to both ADAR1 expression and the ISG score, with a positive interaction term (p for interaction = 0.004)
(JPG)

S4 Fig Compatible levels of editing between Ribo-seq and RNA-seq data. Levels of A-to-I editing in left ventricle DCM patients from RNA-seq (n = 37) and Ribo-seq (n = 30) datasets. Cutoff of ≥ 10 reads for each site.
(JPG)

S5 Fig Comparison of clinical and potential cardiac injury markers by ROC curves. (JPG)

S1 Table Clinical characteristics of GTEx donors. (XLSX)

S2 Table Overview of the analyzed datasets. (XLSX)

S3 Table Significant alterations in the levels of coding editing sites in disease. (CSV)

S4 Table Tables with raw data used for figure plotting. (XLSX)
