# Supplementary figures and images for: Increased A-to-I RNA editing in atherosclerosis and cardiomyopathies
Source: PLoS Comput Biol. 2023 Apr 10;19(4):e1010923. doi: 10.1371/journal.pcbi.1010923 (PMC10085048; doi:10.1371/journal.pcbi.1010923)

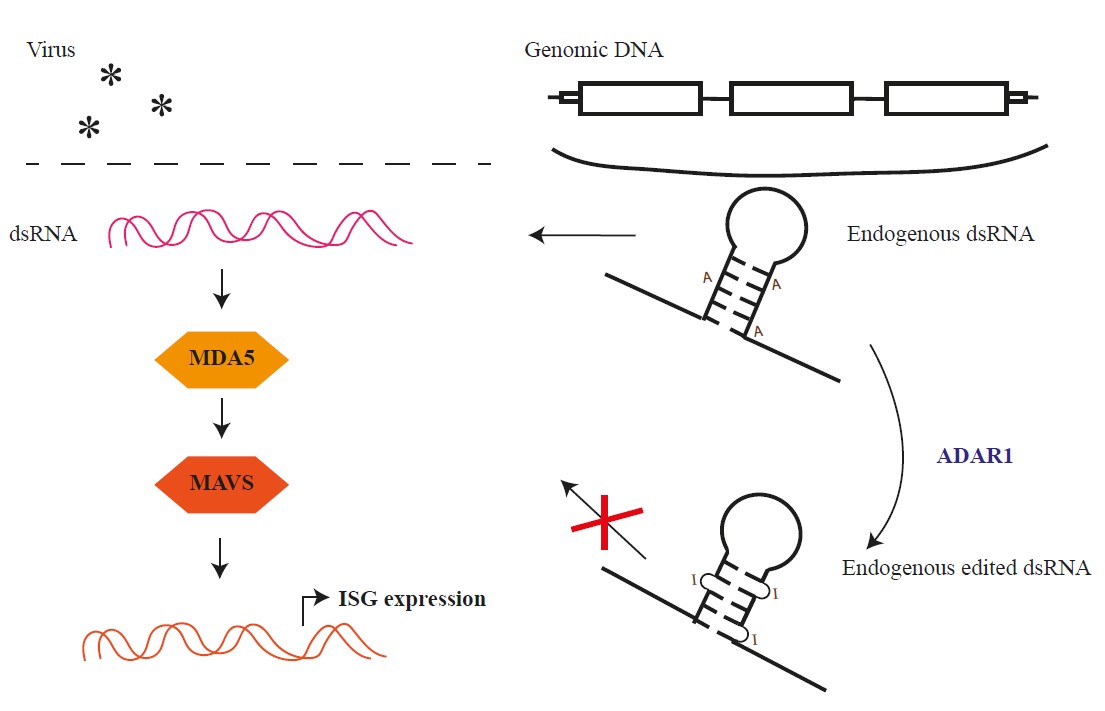

Supplement: S1 Fig — Endogenous dsRNA structures are formed naturally and may activate the inflammation pathway. To prevent false activation of the immune system, ADAR1 disrupts endogenous dsRNA structures by editing adenosines to inosines. Mitochondrial Antiviral Signaling (MAVS); Melanoma Differentiation-Associated protein 5 (MDA5); Interferon Stimulated Genes (ISG); Adenosine Deaminase Acting on RNA 1 (ADAR1). (JPG) [file pcbi.1010923.s001.jpg]

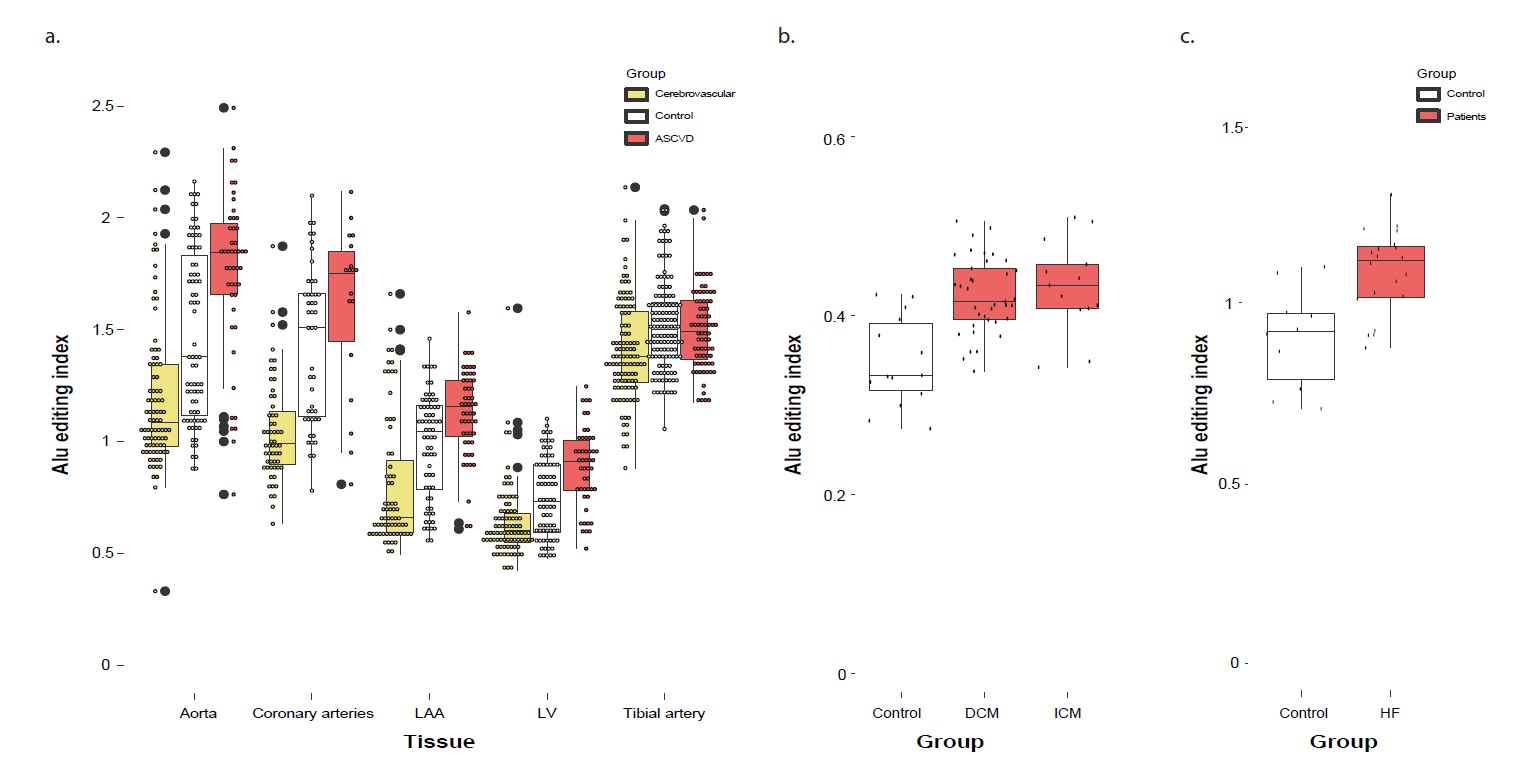

Supplement: S2 Fig — The AEI demonstrates consistent increased editing levels of all Alu sequences in (a) ASCVD patients, and hypo-editing in cerebrovascular patients (Two-sided Wilcoxon rank-sum test, p. value = 0.0004, 0.024, 0.005, 0.0002, 0.78 for ASCVD and 1.7e-6, 4.1e-7, 4.1e-5, 8.6e-5, 0.0001 for cerebrovascular in the aorta, coronary arteries, LAA, LV and tibial artery, respectively). Increased editing levels are also observed in (b) CMP (Two-sided Wilcoxon rank-sum test, p. value = 4e-06 and 1.1e-05 for DCM and ICM, respectively) and (c) ICM (Additional validation set) (Two-sided Wilcoxon rank-sum test, p. value = 0.001). Note that due to differences in read length, the nominal index values cannot be compared between the two panels. (JPG) [file pcbi.1010923.s002.jpg]

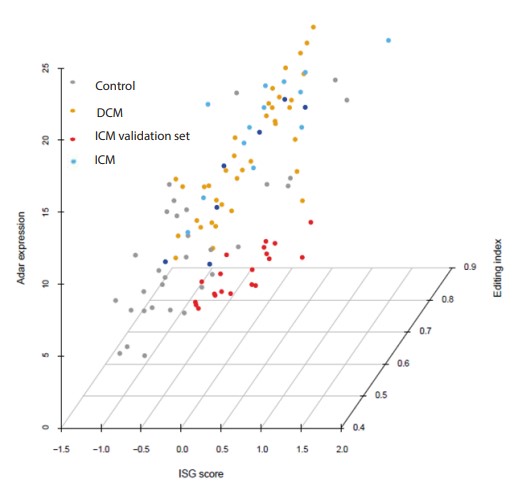

Supplement: S3 Fig — Spearman correlation rho = 0.48, 0.58 and 0.45; p-value = 1.5e-06, 1.2e-09 and 5.9e-06, for ADAR1 expression vs. ISG score, ADAR1 vs. Alu editing and Alu editing vs. ISG, respectively). Linear regression analysis revealed that the Alu editing is significantly correlated to both ADAR1 expression and the ISG score, with a positive interaction term (p for interaction = 0.004) (JPG) [file pcbi.1010923.s003.jpg]

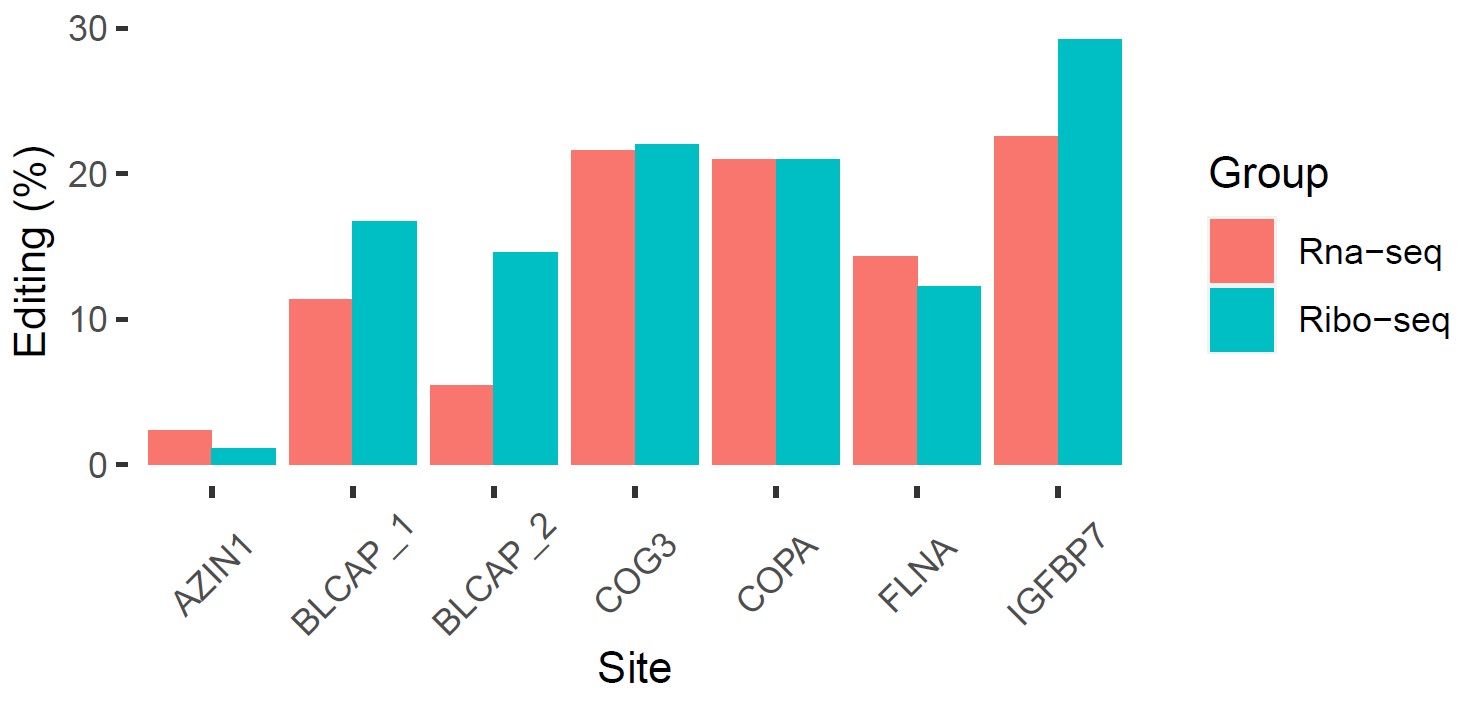

Supplement: S4 Fig — Levels of A-to-I editing in left ventricle DCM patients from RNA-seq (n = 37) and Ribo-seq (n = 30) datasets. Cutoff of ≥ 10 reads for each site. (JPG) [file pcbi.1010923.s004.jpg]

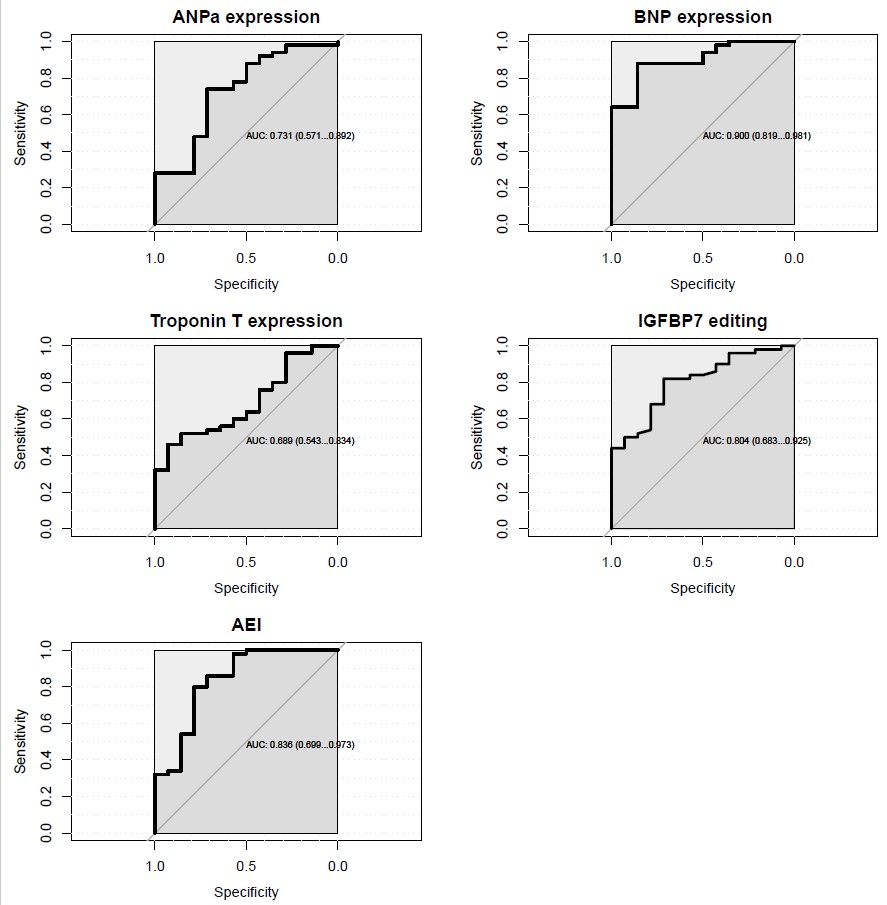

Supplement: S5 Fig — (JPG) [file pcbi.1010923.s005.jpg]
